# Improvement of Cardiac Vegetations in Antiphospholipid Syndrome with Enoxaparin and Corticosteroids after Rivaroxaban Failure

**DOI:** 10.1155/2018/8097539

**Published:** 2018-09-09

**Authors:** Eric Granowicz, Kiyon Chung

**Affiliations:** ^1^Scripps Mercy Hospital, Department of Internal Medicine, San Diego, CA, USA; ^2^Scripps Mercy Hospital, Department of Cardiology, San Diego, CA, USA

## Abstract

Cardiac disease is a well-known complication of antiphospholipid syndrome (APS), with many patients presenting with valvular thickening or vegetations, referred to as Libman–Sacks endocarditis (LSE). Because cases of APS with cardiac involvement are relatively rare, paucity of large clinical trials studying this complication has made management challenging. In the absence of acute heart failure and embolic events, a medical approach is usually selected, consisting of anticoagulation and possibly corticosteroids when another underlying autoimmune disease is present. However, the role of various anticoagulant classes and the duration of steroid therapy continue to be debated. Here, we present a 45-year-old woman who developed two vegetations in the setting of secondary APS while taking rivaroxaban before experiencing marked improvement with the use of enoxaparin and steroids.

## 1. Introduction

Antiphospholipid syndrome (APS) is an autoimmune disease characterized by the presence of various antiphospholipid antibodies that lead to inappropriate activation of platelets and the coagulation cascade. This ultimately manifests clinically with clotting in the venous and/or arterial systems. APS is often secondary to another autoimmune disease such as systemic lupus erythematosus (SLE), or may be a primary phenomenon in the absence of other immunological conditions. In order to make a diagnosis, clinical features must be accompanied by the presence of at least one of three antibodies: anticardiolipin, anti-*β*2 glycoprotein, or lupus anticoagulant.

In some cases, clotting can result in the formation of vegetations on cardiac valves, a form of nonbacterial thrombotic endocarditis (NBTE) called Libman–Sacks endocarditis (LSE). The disease is characterized pathologically by the presence of small, warty lesions of platelets and fibrin usually measuring about 1–5 mm, occurring mainly on the mitral valve, followed by the aortic valve in frequency [1]. The disease ranges from being asymptomatic to presenting with life-threatening signs of systemic emboli or acute heart failure associated with valvular obstruction or regurgitation [2].

Treatment of LSE has been controversial, largely due to the lack of adequate randomized clinical trials. A surgical approach is typically used when criteria similar to those for infectious endocarditis (IE) are met [3–5]. When a medical approach is chosen, treatment depends on whether LSE occurs as a consequence of primary APS, or secondary APS associated with another underlying autoimmune condition. In the former, anticoagulation with warfarin is typically thought to be most effective, with the role of newer oral anticoagulants being less clear [6–8]. In the latter, anticoagulation along with steroid therapy are often used, although some observations have suggested that extended or recurrent use of steroids may actually worsen valvular function over time by leading to excessive scaring and fibrosis [9, 10].

Here, we describe a case of LSE occurring in the setting of secondary APS with SLE in which an abnormally large vegetation formed on the aortic valve concurrently with a smaller vegetation on the mitral valve.

## 2. Case Report

The patient is a 43-year-old female who presented with sharp, substernal, nonexertional chest pain, and shortness of breath. Her past medical history included secondary APS (lupus anticoagulant positive) in the setting of SLE, with multiple, recurrent deep venous thromboses of the lower extremities. She was diagnosed with myopericarditis during a hospitalization one year prior when she presented with similar symptoms and an elevated troponin, after which a coronary catheterization demonstrated no significant coronary artery disease. Examination revealed a 2/6 systolic ejection murmur, without any radiation, gallops, rubs, or jugular venous distension. Auscultation of the lungs revealed clear breath sounds.

**Figure 1:**
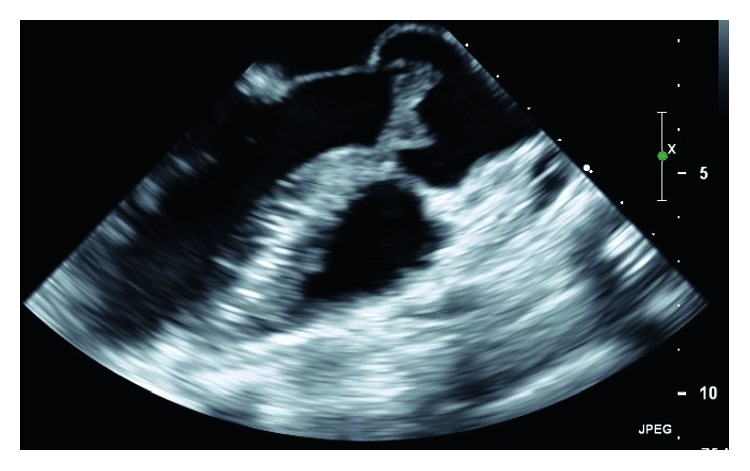
Initial echocardiogram showing a 2 cm mass on the right and noncoronary cusps of the aortic valve.

An echocardiogram was ordered when she was found to have an elevated troponin level of 0.209 ng/ml without any evidence of acute ischemic pathology on her electrocardiogram. Subsequently, a 2 cm mass was seen on the aortic valve with evidence of obstructive pathology. A follow-up transesophageal echocardiogram redemonstrated this mass, along with a smaller mass on the mitral valve associated with mild mitral regurgitation (Figure 1). The aortic mass was consistent with fresh mobile thrombus, somewhat atypical for Libman–Sacks vegetations which usually have a more verrucous appearance. However, after blood cultures and an extensive workup for culture-negative endocarditis were negative, she was ultimately diagnosed with LSE and a recurrent flare of myopericarditis.

**Figure 2:**
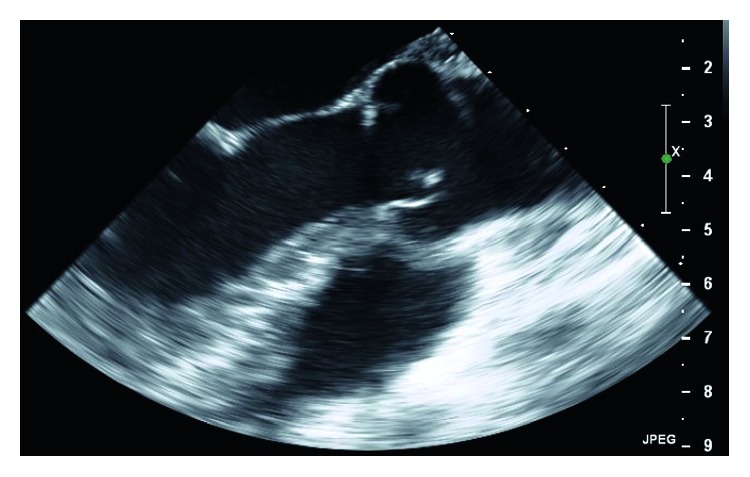
Follow-up transesophageal echocardiogram at 24 weeks showing a dramatic reduction in the size of the mass between the right and noncoronary cusps.

The patient's chest pain improved with colchicine, but her shortness of breath remained and was presumed to be from partial obstruction of the aortic valve by the large mass. After consultation with cardiothoracic surgery, medical therapy was initiated with a goal to avoid surgery if there were signs of improvement. She was already taking rivaroxaban when she originally presented, given that she had failed warfarin therapy in the past with persistently subtherapeutic INRs and recurrent DVTs, so she was started on therapeutic enoxaparin and aspirin. After a rheumatology consultation, hydroxychloroquine and prednisone were initiated as well. The patient remained stable with no new symptoms or signs of embolic events during her follow-up visits, and repeat transesophageal echocardiograms at 12 and 24 weeks demonstrated improvement in the size of both vegetations (Figure 2). Her prednisone was gradually tapered down over a period of 9 months.

## 3. Discussion

This case highlights many of the challenging aspects of managing patients with cardiac involvement in APS. The optimal treatment approach continues to be poorly defined, largely due to the fact that many recommendations regarding management originate from expert opinion and small observational case studies rather than randomized trials.

A treatment strategy begins with assessing the necessity of a surgical approach. Symptoms of acute heart failure, acute valvular regurgitation/stenosis, or recurrent embolization when recognized are considered to be relatively straightforward indications to consider surgical evaluation, in addition to patients who have failed medical management. Surgery is often offered to patients with mobile, left-sided vegetations greater than 1 cm for infective endocarditis, but this approach is not generalizable for cases of NBTE [4, 5]. Given the lack of embolization history, it seemed reasonable to proceed with an aggressive medical treatment plan before considering surgery.

After a commitment is made to a medical approach, a decision about the best form of anticoagulation is imperative. Warfarin with an INR goal of 2-3 has been considered the gold standard in patients with newly diagnosed APS presenting with their first thrombosis [6–8]. Given the relative ease of taking newer oral anticoagulants (NOACs), the role of these agents in APS has begun to be investigated. The RAPS trial compared the use of the direct oral anticoagulant, rivaroxaban, to moderate intensity anticoagulation with warfarin, demonstrating a noninferior anticoagulant ability with similar peak thrombin concentrations after 6 months, though the clinical implications of this finding were limited by insufficient power of the study despite showing no recurrent thromboses in the rivaroxaban group [11].

Additionally, the proper anticoagulant to use in the setting of warfarin failure continues to be perplexing. Various approaches include using high intensity anticoagulation with an INR goal of 3-4, the addition of aspirin or hydroxychloroquine to a moderate intensity anticoagulation regimen, or switching to a new anticoagulant class despite the lack of evidence to support any benefit of one class over vitamin K antagonists [12–14]. Given this patient's failure of warfarin and rivaroxaban, enoxaparin with aspirin seemed to be the most reasonable alternative. Ultimately a favorable response was seen, although it is unclear whether or not the patient's improvement was attributed to the change in anticoagulation or the addition of steroids.

When another underlying autoimmune process is present, steroids are frequently administered despite controversy over their benefits. Several case reports have demonstrated the ability of steroids to acutely decrease the amount of inflammation and disease activity, leading to rapid improvement in symptoms and imaging of the valves [15–17]. Despite these findings, hesitancy remains when deciding to initiate corticosteroid therapy, as several reports have noted that increases in hypertension, left ventricular hypertrophy, and accelerated atherosclerosis are more frequently seen in hearts treated with steroids making symptoms of heart failure more likely in the long term despite the initial acute improvement in symptoms [10, 18, 19]. The initial study that described these findings was done in 1975, and the duration of steroid treatment was unclear for the 36 patients described, so questions still remain about whether or not the benefits of a shorter course of steroids outweigh the potential long term deleterious effects on cardiac structure and function [9]. Additionally, there remains little evidence to support the use of steroids for primary APS.

## 4. Conclusion

This case demonstrates the challenges in managing a complicated presentation of Libman–Sacks endocarditis in the setting of warfarin and rivaroxaban failure. Anticoagulation with enoxaparin and aspirin in combination with prednisone ultimately lead to dramatic improvement, although questions still remain about whether or not this approach could be extrapolated to similar cases with reliable outcomes.
